# Caution regarding the specificities of pan-cancer microbial structure

**DOI:** 10.1099/mgen.0.001088

**Published:** 2023-08-09

**Authors:** Abraham Gihawi, Colin S. Cooper, Daniel S. Brewer

**Affiliations:** ^1^​ Bob Champion Research & Education Building, Norwich Medical School, University of East Anglia, Norwich NR4 7UQ, UK; ^2^​ Earlham Institute, Norwich Research Park, Colney Lane, Norwich NR4 7UG, UK

**Keywords:** cancer, bacteria, viruses, microbiome, machine learning, contamination

## Abstract

Results published in an article by Poore *et al*. (*Nature*. 2020;579:567–574) suggested that machine learning models can almost perfectly distinguish between tumour types based on their microbial composition using machine learning models. Whilst we believe that there is the potential for microbial composition to be used in this manner, we have concerns with the paper that make us question the certainty of the conclusions drawn. We believe there are issues in the areas of the contribution of contamination, handling of batch effects, false positive classifications and limitations in the machine learning approaches used. This makes it difficult to identify whether the authors have identified true biological signal and how robust these models would be in use as clinical biomarkers. We commend Poore *et al*. on their approach to open data and reproducibility that has enabled this analysis. We hope that this discourse assists the future development of machine learning models and hypothesis generation in microbiome research.

## Most models do not perform any better than models constructed using no information

Poore *et al.* [1] detail the building of cancer type models based on microbial interrogation of TCGA (The Cancer Genome Atlas Program) cancer sequence data (which are predominantly RNA sequencing but with some whole genome sequences). Here, we evaluate these models within the framework of Whalen *et al*. [2] describing common modelling pitfalls, namely: (1) distributional differences, (2) confounding, (3) leaky preprocessing and (4) unbalanced classes.

Following their most stringent decontamination, only five of the 33 one-vs-all cancer type models examined were a statistically significantly improvement on models constructed using no information [at the 0.05 significance level, without false discovery correction for multiple models, ‘*P*-Value (Acc>NIR)’, available: http://cancermicrobiome.ucsd.edu/CancerMicrobiome_DataBrowser/] – this was not clear in the main text of their paper.

## Models pronounce nonsensical genera are informative of tumour type

Even when the model does appear to identify samples better than the negative predictor, we have concerns that many of the key features used in the model are implausible. For example, the model predicting adrenocortical carcinoma is significantly better than a negative predictor (*P*=0.002) and boasts high sensitivity (0.9565), specificity (0.998) and positive predictive values (0.71). Therefore, this model should hold some features that truly distinguish it from the remaining cancer types. The top ten most important features for this model are *Hepandensovirus* (relative feature importance score: 9431, a virus that infects crustaceans [3]), *Paeniclostridium* (973), *Comovirus* (846), *Thalassomonas* (267, bacteria causing coral disease [4]), *Simkania* (160), *Cronobacter* (151), *Simonsiella* (148), *Leucothrix* (145, bacteria from marine macroalgae [5]), *Phikmvlikevirus* (128) and *N4likevirus* (88). It is unclear how *Phikmvlikevirus* and *N4likevirus* might be informative for adrenocortical carcinoma as they are bacteriophages and therefore would be dependent on the co-occurrence of their bacterial hosts in the adrenal glands (or alternatively the remaining anatomical locations [6, 7]). Many of the top performing features of other models under the most stringent decontamination approach also seem nonsensical (Table 1). This point is not covered by the Whalen pitfalls because it is generally presumed that the features being modelled exist to begin with, which in the case of taxonomic classification is not always true.

**Table 1.** Top performing features for a selection of one-vs-all cancer type models in the most stringent decontamination approach as presented in Poore *et al* These taxa include extremophiles that have not previously been isolated from humans. See Table S1, available in the online version of this article (bacteria), and Table S2 (viruses) for a full description as on NCBI of the sources for each representative species within these genera.

Genus	Top feature in cancer type model	Details
* Leucothrix *	Bladder cancer	Bacteria from marine macroalgae [5]
* Thalassomonas *	Uveal melanoma	Bacteria causes disease in coral [4]
*Velarivirus*	Cervical cancer	Grapevine is natural host [33]
*Tritimovirus*	Colon cancer	Known to infect cereals [34]
*Dinovernavirus*	Renal clear cell carcinoma	Contains insect viruses [35]
*Bacillarnavirus*	Lung squamous cell carcinoma	Infects algae [36]
*Rymovirus*	Ovarian serous	Infects species of grass [37]
* Ignicoccus *	Prostate	Identified in marine hydrothermal vents [19]
* Salinimicrobium *	Testicular cancer	Halophilic genus identified from marine environments [38]

Some models do demonstrate plausible and promising results. For example, in hepatocellular carcinoma, *Orthohepadnavirus* is known to have a causal relationship with cancer formation [8] and has been found to be specific to the liver in other datasets [9]. This is reflected well in Poore *et al.*’s model where the estimated variable importance score of *Orthohepadnavirus* in their model (2020.53) dwarfs the next most ‘important’ feature (*Levivirus*, 975.09). Despite this, the model is still not significantly better than a negative predictor [*P*-value (Acc>NIR)=1] and suffers a poor positive predictive value (0.4).

## Potential for read misclassification

We believe that these nonsensical genera arise because the models produced by Poore *et al*. are built on many features that are likely to be taxonomically misclassified, from human reads or other contamination [10–14], and therefore do not originate from microbes in the sample. One possible reason for these misclassifications is that extra steps were not taken to remove human reads prior to model building. Poore *et al.* detail the extraction of reads unaligned to a human reference genome which are then the subject of taxonomic classification. The authors claim to have used ‘very stringent decontamination analyses that discarded up to 92.3 % of total sequence data’. This would suggest that 7.7 % of all sequencing reads were subject to taxonomic classification. This pool of reads will still contain human reads which have not aligned [15]. For example, this could be because the reads are of low quality or they are mutated in cancer genomes, or due to sequencing artefacts. In addition, the authors detail no human reference sequences in their taxonomic database, using 59 974 microbial genomes only. Therefore, it is highly likely that human sequences will have been misclassified as microbial. The subsequent application of SHOGUN alignment of Kraken-classified reads is more specific but may still involve the inappropriate classification of human reads to a database with no representation of the human genome. Additional human depletion filtering and steps to remove contamination such as those employed by the cancer microbiome atlas to distinguish tissue-resident microbiota from contaminants would have helped to remove misclassifications [16].

## Normalization introduces variance and permits modelling

**Fig. 1.**
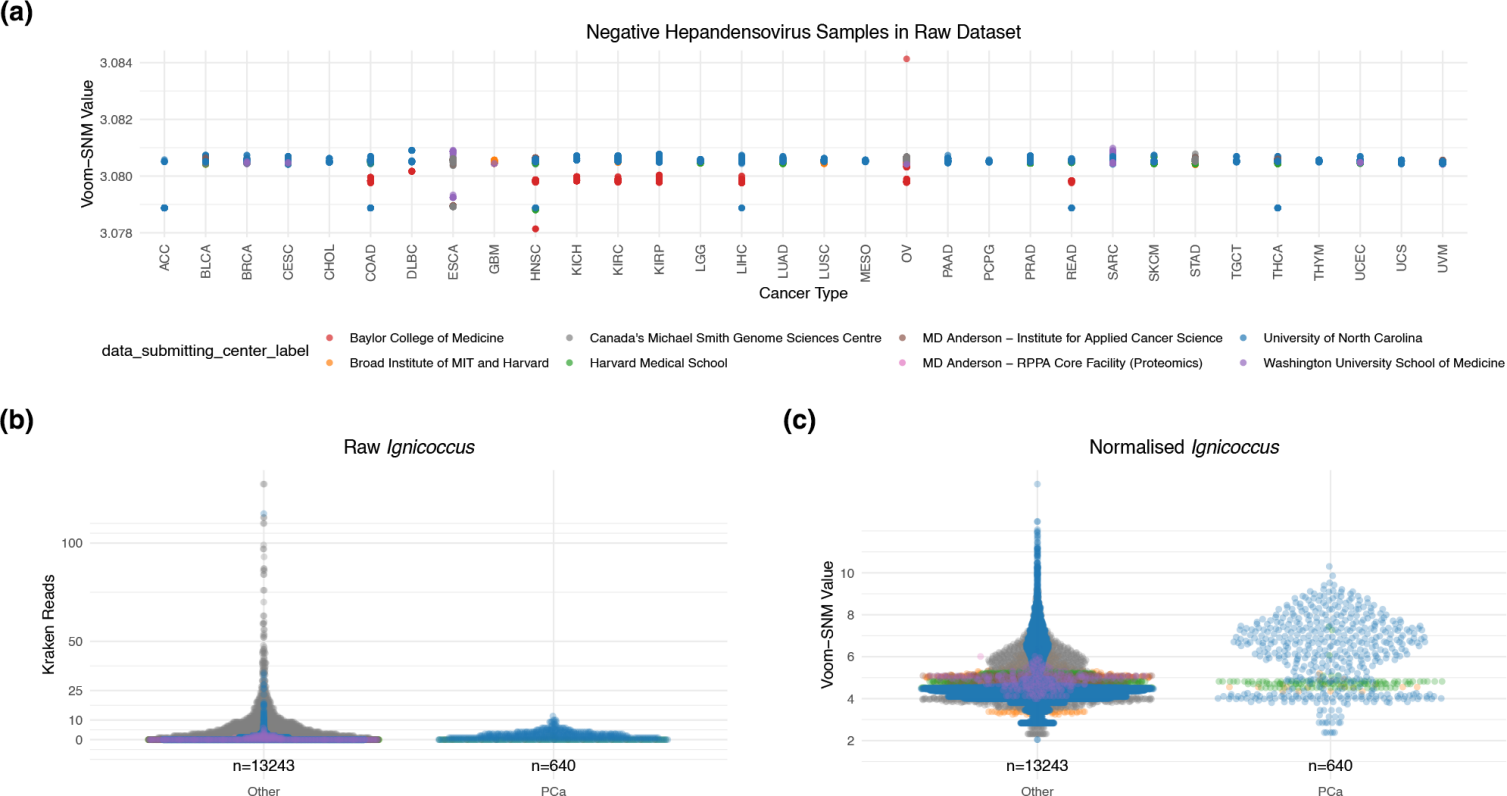
(a) Voom-SNM normalized TCGA samples (*n*=17 624) that were negative for crustacean virus hepandensovirus with zero classified reads in the original Kraken dataset with the most stringent decontamination approach. One sample contained two sequencing reads for *Hepandensovirus*, which has been omitted from this figure to illustrate inappropriate variation introduced by SNM. The colour of each point indicates the centre where the sample was sequenced and from where the resulting data were submitted [University of North Carolina, Harvard Medical School, Canada’s Michael Smith Genome Sciences Centre, Broat Institute MIT and Harvard, Baylor College of Medicine, Washington University School of Medicine, MD Anderson – Institute for Applied Cancer Science, Johns Hopkins/University of Southern California, MD Anderson RPPA Core Facility (Proteomics)]. The *x*-axis demonstrates cancer types using TCGA abbreviations as in Poore *et al.* [1]. This is a prominent concern, especially given how closely linked sequencing centre and disease type are (Table S3). Raw (**b**) and Voom-SNM normalized (**c**) *Ignicoccus* values, which was deemed the most important feature for predicting prostate cancer (PCa) from all other cancer types (*n*=13 883 primary tumours). Median values are as follows: Kraken raw other 0, Kraken raw PCa 1, normalized other 4.49, normalized PCa 5.82. In both the raw and normalized cases, the distributions are significantly different (Wilcox signed rank-sum test *P*<2.2×10^–16^).

Another possible contributing factor to the issues with the models is in how the data were processed. Microbiome data are dynamic [17] (Whalen I: distributional differences), and are typically heteroskedastic (meaning that the variance of a variable is non-constant over values of an independent variable, i.e. the number of sequencing reads assigned to each of two genera) [18]. The authors resolve heteroskedasticity by applying a tool called Voom that is designed for RNA sequencing data of a single organism where the majority of genes have some level of expression. However, as applied by Poore *et al.* it suggests presence even when taxa are absent (Whalen III: leaky preprocessing). For example, for *Hepandensovirus* (genus of crustacean virus), the top feature for adrenocortical carcinoma, Voom transitions all zeros to non-zero values and untrue variation has been introduced by the global adjustment for technical variables including sequencing centre (Fig. 1a, batch correction relating to Whalen II: confounding). Therefore, this normalization appears beneficial on the global level but raises prominent concerns at the level of individual taxa.

Another example of how the processing of data can be problematic is provided by the extremophile genus *Ignicoccus* in prostate cancer samples. *Ignicoccus* shows a statistically significant increase in prostate cancer samples compared to other cancers in the normalized dataset (Wilcox signed rank-sum test *P*<2.2×10^–16^, Fig. 1b, c). In the raw, unprocessed data no increase in prostate cancer samples is apparent. Indeed, most values are zero and the maximum number of reads found in the raw prostate cancer data for *Ignicoccus* is 12 (low evidence of detection). It is also highly likely that these are false taxonomic assignments given that *Ignicoccus* was identified in marine hydrothermal vents [19]. This taxon should have been filtered out prior to model building – the application of a minimum read threshold (i.e. 100 classified reads) would have assisted the removal of spurious taxa.

## The models are trained on unbalanced data

The performance of the models may in part be due to the major imbalance in class size in the datasets (Whalen IV: unbalanced classes), meaning that before model construction, data in the cancer set under investigation are multiplied up many times (upsampling) so that patient numbers in the ‘cancer groups’ and in the ‘all other cancers group’ become similar. This approach may amplify the prominence of implausible artefactual data. Adrenocortical carcinoma for example has 79 associated samples (as per Metadata-TCGA-All-18116-Samples.csv provided by Poore *et al.*). This means that 18 037 are not adrenocortical carcinoma. Adrenocortical carcinoma therefore represents 0.44 % of the whole dataset and therefore data from adrenocortical carcinoma are amplified up to 230 times to equal the sample size of the rest of the dataset. The modelling is therefore overexposed to inappropriate variation in taxa such as *Hepandensovirus* (Fig. 1).

## Discussion

The detection of microbial composition via machine learning is increasingly being used in disease-based research. Extreme caution must be taken to avoid coming to inaccurate conclusions. In this letter we have reviewed the paper of Poore *et al.* [1] and highlighted many problems. Ideally, the authors would have followed as closely as possible the RIDE criteria set out by Eisenhofer *et al.* (also authored by Knight) [20]. Where this is not possible, the conclusions drawn should be more cautious and the limitations made clear. Poore *et al.* use many good practices in machine learning [21] but there is the need to avoid the pitfalls of Whalen *et al.* and use more stringent methods regarding contamination, taxonomic misclassification and previous microbiological evidence.

The hypothesis that microbes (including those found in tumours) are dependent on anatomical location is well founded based on previous work [22], but the models produced by Poore *et al.* are at best suggestive and do not substantiate this observation. Additional care should be taken to include only taxa with strong evidence of presence based on computational evidence, consideration of the likelihood of contamination and prior biological evidence that the taxa are present in the biological sample of interest.

After we raised our concerns [23], Poore *et al.* published a response to our points [24]. Despite its considerable length, it focused on the technical details of statistical modelling and did not address the core concerns raised in this letter regarding contamination, nonsensical taxa appearing as important and the flawed batch correction.

Even in the technical areas where they did respond, they did not address these points. For example, Poore *et al.* defended the use of Voom prior to batch correction by claiming that it had been cited >4000 times, but Voom was not developed for metatranscriptomics. Microbial community matrices are typically much sparser than single-organism gene expression matrices. Voom transforms zero values into non-zero values, subsequently with additional false signal introduced (Fig. 1a), which makes the use case of Voom followed by SNM inappropriate. In their response, they suggest that a difference of 0.006 in the normalized values for *Hepandensovirus* in adrenocortical carcinoma (Fig. 1a) is not significant. This is not correct. The machine learning algorithms used in Poore *et al.* do not require large differences to build a rule and make classifications. This is reflected in the fact that this genus is by far the most important feature in their near perfect performing model for predicting adrenocortical carcinoma.

The overarching problem, however, is the prevalence of nonsensical taxa appearing as informative in Poore *et al.*’s models. This is a sure sign that something is going wrong. Poore *et al.* have given some attention to the issue of contamination but nonsensical taxa with limited evidence of true involvement are still prominent, suggesting this has not gone far enough. In their response, Poore *et al.* also state that they ‘extensively remove human reads from metagenomic data’, but sequencing reads that are not aligned to the human genome are not equivalent to non-human reads and there is no evidence that a human genome was present in their taxonomic database, which is best practice [25]. Poore *et al.* noted that the most stringent decontaminated dataset was only produced to address a reviewer’s concerns but that the structure of the data soon became unrecognizable. It is therefore alarming that performance metrics are still high and that nonsensical taxa are still reported as the best performing features in the models built on these ‘unrecognizable’ datasets with ‘stringent’ decontamination. Contamination is undoubtedly a major concern in microbiome research and has critically affected the results of a significant amount of research [10, 11, 13, 14, 20]. Examples include the claims of a brain or placental microbiome [11, 12].

It is our contention that there are critical flaws in the study by Poore *et al.* resulting in misclassifications and contamination being considered as important features to predict tumour type. Unless this issue is addressed, no matter how good the subsequent analysis, the results will still be questionable. Therefore, we believe that our central point of urging ‘caution’ to those interpreting the data and results of Poore *et al.* remains valid.

Finally, we would like to highlight the controversy surrounding the use of the term ‘cancer microbiome’ in this context. There are many definitions of ‘microbiome’ [26], but the commonly accepted use of the term could imply that microbes are ubiquitous in every single cancer sample, which they are not. There are many sites in the body with highly disproven ‘microbiomes’ such as the uterus and brain [11–14]. Given the methodological issues we raise, it is difficult to see whether any of the reported microbes are cancer type specific or whether they go beyond the known tissue-specific microbes (hepatitis etc.). Therefore, it should be considered whether these really constitute a ‘microbiome’ or whether they are related to infection.

## Conclusion

We believe that the study of microbes in tumours is an exciting field, and that the use of large sequencing datasets with rich metadata can reveal much more about the nature of the interplay between microbes and cancer. Poore *et al.* have used machine learning models to describe the ‘tumour microbiome’ as being specific to tumour type, but we have serious concerns. Overwhelming contamination and inappropriate handling of the data do not support the claims in the original title: ‘Microbiome analyses of blood and tissues suggest cancer diagnostic approach’. A dataset with a less pronounced batch effect, more balanced class sizes and modelling all tumour types at once (not one-vs-all models) might help to better distinguish the pan-cancer microbial structure. There needs to be a better demonstration of microbial differences between tumour types and rigorous validation of models before we can be certain of these differences and illuminate any taxa underpinning these differences. We are a long way from proving the utility of cancer microbial structure in improving cancer patient care.

## Methods

All analysis in this paper was conducted on the open-source data made available by Poore *et al.* [1] available at: http://ftp.microbio.me/pub/cancer_microbiome_analysis/. Files analysed include: Kraken-TCGA-Raw-Data-17625-Samples.csv (MD5 checksum: 6af81818f69bf56b79836e1c317c3e03), Metadata-TCGA-All-18116-Samples.csv (MD5 checksum: dbdd1f64d45973977fc8435db2eb8b3e), and Kraken-TCGA-Voom-SNM-Most-Stringent-Filtering-Data.csv (MD5 checksum: b7e50700b791b8881426aeb1fa12c3bb).

Model performance and feature importance was accessed: http://cancermicrobiome.ucsd.edu/CancerMicrobiome_DataBrowser/. All data were analysed in R (version 4.2.1). Packages used include tidyverse [27] (version 1.3.2), ggpubr [28] (version 0.5.0), ggbeeswarm [29] (version 0.7.1), cowplot [30] (version 1.1.1) and EnvStats [31] (version 2.7.0). Hypothesis testing was performed with the wilcox.test() function.

Representative species within top features (Table S1) were identified by browsing GTDB [32] (release version 207). Associated metadata regarding isolation sources were found by accessing links presented on the GTDB taxonomy browser.

## Supplementary Data

Supplementary material 1

## References

[1] Poore GD, Kopylova E, Zhu Q, Carpenter C, Fraraccio S (2020). Microbiome analyses of blood and tissues suggest cancer diagnostic approach. Nature.

[2] Whalen S, Schreiber J, Noble WS, Pollard KS (2022). Navigating the pitfalls of applying machine learning in genomics. Nat Rev Genet.

[3] Cotmore SF, Agbandje-McKenna M, Chiorini JA, Mukha DV, Pintel DJ (2014). The family Parvoviridae. Arch Virol.

[4] Hosoya S, Adachi K, Kasai H (2009). *Thalassomonas actiniarum* sp. nov. and *Thalassomonas haliotis* sp. nov., isolated from marine animals. Int J Syst Evol Microbiol.

[5] Liu T, Zhang Y, Zhang X, Zhou L, Meng C (2019). *Leucothrix sargassi* sp. nov., isolated from a marine alga [*Sargassum natans* (L.) Gaillon]. Int J Syst Evol Microbiol.

[6] Wittmann J, Klumpp J, Moreno Switt AI, Yagubi A, Ackermann H-W (2015). Taxonomic reassessment of N4-like viruses using comparative genomics and proteomics suggests a new subfamily - “Enquartavirinae.”. Arch Virol.

[7] Merabishvili M, Vandenheuvel D, Kropinski AM, Mast J, De Vos D (2014). Characterization of newly isolated lytic bacteriophages active against *Acinetobacter baumannii*. PLoS One.

[8] Ringelhan M, McKeating JA, Protzer U (2017). Viral hepatitis and liver cancer. Philos Trans R Soc Lond B Biol Sci.

[9] Zapatka M, Borozan I, Brewer DS, Iskar M, Grundhoff A (2020). The landscape of viral associations in human cancers. Nat Genet.

[10] Salter SJ, Cox MJ, Turek EM, Calus ST, Cookson WO (2014). Reagent and laboratory contamination can critically impact sequence-based microbiome analyses. BMC Biol.

[11] de Goffau MC, Lager S, Sovio U, Gaccioli F, Cook E (2019). Human placenta has no microbiome but can contain potential pathogens. Nature.

[12] Bedarf JR, Beraza N, Khazneh H, Özkurt E, Baker D (2021). Much ado about nothing? Off-target amplification can lead to false-positive bacterial brain microbiome detection in healthy and Parkinson’s disease individuals. Microbiome.

[13] de Goffau MC, Lager S, Salter SJ, Wagner J, Kronbichler A (2018). Recognizing the reagent microbiome. Nat Microbiol.

[14] de Goffau MC, Charnock-Jones DS, Smith GCS, Parkhill J (2021). Batch effects account for the main findings of an in utero human intestinal bacterial colonization study. Microbiome.

[15] Gihawi A, Rallapalli G, Hurst R, Cooper CS, Leggett RM (2019). SEPATH: benchmarking the search for pathogens in human tissue whole genome sequence data leads to template pipelines. Genome Biol.

[16] Dohlman AB, Arguijo Mendoza D, Ding S, Gao M, Dressman H (2021). The cancer microbiome atlas: a pan-cancer comparative analysis to distinguish tissue-resident microbiota from contaminants. Cell Host Microbe.

[17] Gerber GK (2014). The dynamic microbiome. FEBS Lett.

[18] McMurdie PJ (2018). Normalization of microbiome profiling data. Methods Mol Biol.

[19] Paper W, Jahn U, Hohn MJ, Kronner M, Näther DJ (2007). *Ignicoccus hospitalis* sp. nov., the host of “Nanoarchaeum equitans.”. Int J Syst Evol Microbiol.

[20] Eisenhofer R, Minich JJ, Marotz C, Cooper A, Knight R (2019). Contamination in low microbial biomass microbiome studies: issues and recommendations. Trends Microbiol.

[21] Knight R, Vrbanac A, Taylor BC, Aksenov A, Callewaert C (2018). Best practices for analysing microbiomes. Nat Rev Microbiol.

[22] Costello EK, Lauber CL, Hamady M, Fierer N, Gordon JI (2009). Bacterial community variation in human body habitats across space and time. Science.

[23] Gihawi A, Cooper CS, Brewer DS (2023). Caution regarding the specificities of pan-cancer microbial structure. Bioinformatics.

[24] Sepich-Poore GD, Kopylova E, Zhu Q, Carpenter C, Fraraccio S (2023). Reply to: caution regarding the specificities of pan-cancer microbial structure. Bioinformatics.

[25] Breitwieser FP, Lu J, Salzberg SL (2019). A review of methods and databases for metagenomic classification and assembly. Brief Bioinform.

[26] Berg G, Rybakova D, Fischer D, Cernava T, Vergès M-CC (2020). Microbiome definition re-visited: old concepts and new challenges. Microbiome.

[27] Wickham H, Averick M, Bryan J, Chang W, McGowan L (2019). Welcome to the Tidyverse. J Open Source Softw.

[28] Kassambara A (2022). ggpubr: “ggplot2” Based Publication Ready Plots.

[29] Clarke E-M, ggbeeswarm C (2022). Categorical Scatter (Violin Point) Plots.

[30] Wilke C (2020). cowplot: Streamlined Plot Theme and Plot Annotations for 'ggplot2.

[31] Millard SP (2013). EnvStats: An R Package for Environmental.

[32] Parks DH, Chuvochina M, Rinke C, Mussig AJ, Chaumeil PA (2022). GTDB: an ongoing census of bacterial and archaeal diversity through a phylogenetically consistent, rank normalized and complete genome-based taxonomy. Nucleic Acids Res.

[33] Yu H, Qi S, Chang Z, Rong Q, Akinyemi IA (2015). Complete genome sequence of a novel velarivirus infecting areca palm in China. Arch Virol.

[34] Rabenstein F, Seifers DL, Schubert J, French R, Stenger DC (2002). Phylogenetic relationships, strain diversity and biogeography of tritimoviruses. J Gen Virol.

[35] Roundy CM, Azar SR, Rossi SL, Weaver SC, Vasilakis N (2017). Insect-specific viruses: a historical overview and recent developments. Adv Virus Res.

[36] Short SM, Staniewski MA, Chaban YV, Long AM, Wang D (2020). Diversity of viruses infecting eukaryotic algae. Curr Issues Mol Biol.

[37] Webster DE, Beck DL, Rabenstein F, Forster RLS, Guy PL (2005). An improved polyclonal antiserum for detecting Ryegrass mosaic rymovirus. Arch Virol.

[38] Nedashkovskaya OI, Vancanneyt M, Kim SB, Han J, Zhukova NV (2010). *Salinimicrobium marinum* sp. nov., a halophilic bacterium of the family Flavobacteriaceae, and emended descriptions of the genus *Salinimicrobium* and *Salinimicrobium catena*. Int J Syst Evol Microbiol.

