# Supplementary material for: Caution regarding the specificities of pan-cancer microbial structure
Source: Microb Genom. 2023 Aug 9;9(8):mgen001088. doi: 10.1099/mgen.0.001088 (PMC10483429; doi:10.1099/mgen.0.001088)
Supplement: Supplementary material 1 [file mgen-9-1088-s001.pdf]

Supplementary Table 1 - These species were found on GTDB taxonomy and include species representatives that passed quality checks. Each of these species originates from a genus that was the top performing microbial feature in the one-vs-all tumour models provided by Poore *et al*. [1] and was considered the most stringent approach to decontamination.

| GTDB Taxonomy | Isolation Source Information |
|---|---|
| Leucothrix sp002747455 | swab sample of gingival sulcus (mouth) from 29 year old lactating female Dolphin_Z https://www.ncbi.nlm.nih.gov/biosample/SAMN07757601/ |
| Leucothrix mucor | seaweed, Friday Harbor, WA https://www.ncbi.nlm.nih.gov/biosample/SAMN02440559/ |
| Leucothrix pacifica | Seawater - Pacific Ocean: South Pacific Gyre https://www.ncbi.nlm.nih.gov/biosample/SAMN09228920/ |
| Leucothrix arctica | Seawater - Svalbard: coast of Kongsfjorden, Arctic https://www.ncbi.nlm.nih.gov/biosample/SAMN09228919/ |
| Thalassomonas actiniarum | Sea anemone<br><br>https://www.ncbi.nlm.nih.gov/biosample/SAMN03278135/ |
| Thalassomonas viridans | Cultivated oysters https://www.ncbi.nlm.nih.gov/biosample/SAMN03278134/ |
| Ignicoccus_A sp013154085 | deepsea hydrothermal sulfide chimney https://www.ncbi.nlm.nih.gov/biosample/SAMN12405658/ |
| Ignicoccus_A sp015521275 | marine hydrothermal vent biome https://www.ncbi.nlm.nih.gov/biosample/SAMN12837294/ |
| Ignicoccus_A hospitalis | ***Ignicoccus* hospitalis Kin4/I**. *Ignicoccus* hospitalis Kin4/I was isolated from gravel obtained from the shallow marine hydrothermal system of the Kolbeinsey Ridge, north of Iceland and will be used for comparative analysis with other thermophiles https://www.ncbi.nlm.nih.gov/bioproject/13914 |
| Ignicoccus islandicus | "The genome sequence of this strain will provide information on the adaptation to high temperature environments and be used for evolutionary and comparative genomic analysis." https://www.ncbi.nlm.nih.gov/bioproject/PRJNA61347/ |
| Salinimicrobium xinjiangense | Soil sediment of a salt lake https://www.ncbi.nlm.nih.gov/biosample/SAMN02440840/ |
| Salinimicrobium terrae | Saline soil https://www.ncbi.nlm.nih.gov/biosample/SAMN02440676/ |
| Salinimicrobium sp012038135 | marine sediment<br><br>https://www.ncbi.nlm.nih.gov/biosample/SAMN14480397/ |
| Salinimicrobium marinum | Unstated https://www.ncbi.nlm.nih.gov/biosample/SAMD00245570/ |
| Salinimicrobium catena | Unstated https://www.ncbi.nlm.nih.gov/biosample/SAMN04488140/ |
| Salinimicrobium sediminis | Unstated https://www.ncbi.nlm.nih.gov/biosample/SAMN06296241/ |

Supplementary Table 2 – Viruses as top features in one-vs-all models. These were identified by searching NCBI at genus level and investigating each individual species. Each of these species originates from a genus that was the top performing feature in one of the one-vs-all tumour models presented by Poore *et al*. under the most stringent decontamination conditions.

| | |
|---|---|
| *Areca palm velarivirus 1* | Complete genome sequence of a novel Velarivirus infecting areca palm in China https://www.ncbi.nlm.nih.gov/nuccore/NC_027121.1/ |
| *Cordyline virus 1* (velarivirus) | An assemblage of closteroviruses infects Hawaiian ti (Cordyline fruticosa L.) https://www.ncbi.nlm.nih.gov/nuccore/NC_038421.1/ |
| *Cordyline virus* 2 (velarivirus) | Differentiation, distribution, and elimination of closteroviruses infecting Cordyline fruticosa (L.) in Hawaii https://www.ncbi.nlm.nih.gov/nuccore/NC_043453.1/ |
| *Cordyline virus 3* (velarivirus) | Differentiation, distribution, and elimination of closteroviruses infecting Cordyline fruticosa (L.) in Hawaii https://www.ncbi.nlm.nih.gov/nuccore/NC_043107.1/ |
| *Cordyline virus 4* (velarivirus) | Differentiation, distribution, and elimination of closteroviruses infecting Cordyline fruticosa (L.) in Hawaii https://www.ncbi.nlm.nih.gov/nuccore/NC_043108.1/ |
| *Grapevine leafroll-associated virus 7* (velarivirus) | Molecular characterization and taxonomy of grapevine leafroll-associated virus 7 https://www.ncbi.nlm.nih.gov/nuccore/NC_016436.1/ |
| *Little cherry virus 1*<br>*Malus domestica virus A* (velarivirus) | Complete genome structure and phylogenetic analysis of little cherry virus, a mealybug-transmissible closterovirus https://www.ncbi.nlm.nih.gov/nuccore/NC_001836.1/ |
| *Malus domestica virus A* (valarivirus) | Genomic characterization of a novel velarivirus Malus domestica virus A (MdoVA) infecting apple https://www.ncbi.nlm.nih.gov/nuccore/NC_055599.1/ |
| *Brome streak mosaic virus* (***Tritimovirus***) | The complete nucleotide sequence and genome organization of the mite-transmitted brome streak mosaic rymovirus in comparison with those of potyviruses https://www.ncbi.nlm.nih.gov/nuccore/NC_003501.1/ |
| *Oat necrotic mottle virus* (***Tritimovirus***) | Complete nucleotide sequence of Oat necrotic mottle virus: a distinct Tritimovirus species (family Potyviridae) most closely related to Wheat streak mosaic virus https://www.ncbi.nlm.nih.gov/nuccore/NC_005136.1/ |
| *Tall oatgrass mosaic virus* (***Tritimovirus***) | Tall oatgrass mosaic virus (TOgMV): a novel member of the genus Tritimovirus infecting Arrhenatherum elatius https://www.ncbi.nlm.nih.gov/nuccore/NC_022745.1/ |
| *Wheat eqlid mosaic virus* (***Tritimovirus***) | Analyses of the complete sequence of the genome of wheat Eqlid mosaic virus, a novel species in the genus Tritimovirus https://www.ncbi.nlm.nih.gov/nuccore/NC_009805.1/ |
| *Wheat streak mosaic virus* (***Tritimovirus***) | Phylogenetic relationships within the family potyviridae: wheat streak mosaic virus and brome streak mosaic virus are not members of the genus rymovirus https://www.ncbi.nlm.nih.gov/nuccore/NC_001886.1/ |
| *Yellow oat grass mosaic virus* (***Tritimovirus***) | Genome sequence of two isolates of Yellow oatgrass mosaic virus, a new grass-infecting Tritimovirus https://www.ncbi.nlm.nih.gov/nuccore/NC_024471.1/ |
| *Aedes pseudoscutellaris reovirus* (Dinovernavirus) | isolated from Aedes pseudoscutellaris mosquito cells https://www.ncbi.nlm.nih.gov/nuccore/NC_007666.1/ 10.1016/j.virol.2005.08.028 |

| | |
|---|---|
| *Chaetoceros socialis forma radians RNA virus 1* (Bacillarnavirus) | Isolation and characterization of a single-stranded RNA virus infecting the bloom-forming diatom Chaetoceros socialis https://www.ncbi.nlm.nih.gov/nuccore/NC_012212.1/ |
| *Chaetoceros tenuissimus RNA virus 01* (Bacillarnavirus) | Isolation and characterization of a single-stranded RNA virus infecting the marine planktonic diatom Chaetoceros tenuissimus Meunier https://www.ncbi.nlm.nih.gov/nuccore/NC_038321.1/ |
| *Rhizosolenia setigera RNA virus 01* (Bacillarnavirus) | Complete nucleotide sequence of a single-stranded RNA virus infecting the bloom-forming diatom Rhizosolenia setigera https://www.ncbi.nlm.nih.gov/nuccore/NC_018613.1/ |
| *Agropyron mosaic virus* (Rymovirus) | Functional replacement of Wheat streak mosaic virus HC-Pro with the corresponding cistron from a diverse array of viruses in the family https://www.ncbi.nlm.nih.gov/nuccore/NC_005903.1/ |
| *Hordeum mosaic virus* (Rymovirus) | Functional replacement of Wheat streak mosaic virus HC-Pro with the corresponding cistron from a diverse array of viruses in the family Potyviridae https://www.ncbi.nlm.nih.gov/nuccore/NC_005904.1/ |
| *Ryegrass mosaic virus* (Rymovirus) | The complete nucleotide sequence of the Ryegrass mosaic virus https://www.ncbi.nlm.nih.gov/nuccore/NC_001814.1/ |

Supplementary Table 3 – The proportion (and total number) of all primary tumour samples across sequencing centers as provided by Poore et al. in file: Metadata-TCGA-All-18116-Samples.csv. Any cells where at least 90% of the samples originate from one sequencing center are highlighted in red.

| | **Sequencing Center** | | | | | | | |
|---|---|---|---|---|---|---|---|---|
| **Primary Tumour Type** | **Baylor College of Medicine** | **Broad Institute of MIT and Harvard** | **Canada's Michael Smith Genome Sciences Centre** | **Harvard Medical School** | **MD Anderson - Institute for Applied Cancer Science** | **MD Anderson - RPPA Core Facility (Proteomics)** | **University of North Carolina** | **Washington University School of Medicine** |
| **Adrenocortical Carcinoma** | 0 | 0 | 0 | 0 | 0 | 0 | 1 (76) | 0 |
| **Bladder Urothelial Carcinoma** | 0 | 0.05 (27) | 0 | 0.09 (50) | 0.11 (60) | 0 | 0.75 (406) | 0 |
| **Brain Lower Grade Glioma** | 0 | 0.05 (30) | 0 | 0.09 (51) | 0 | 0 | 0.86 (505) | 0 |
| **Breast Invasive Carcinoma** | 0 | 0 | 0 | 0.02 (18) | 0 (1) | 0 | 0.92 (1046 | 0.07 (78) |
| **Cervical and Endocervical** | 0 | 0 | 0 | 0.02 (6) | 0.11 (41) | 0 | 0.82 (293) | 0.05 (18) |

| | | | | | | | | |
|---|---|---|---|---|---|---|---|---|
| **Cholangiocarcinoma** | 0 | 0 | 0 | 0 | 0 | 0 | 1 (36) | 0 |
| **Colon Adenocarcinoma** | 0.05 (40) | 0 | 0 | 0.11 (86) | 0 | 0 | 0.84 (651) | 0 |
| **Esophageal Carcinoma** | 0 | 0 | 0.69 (147) | 0 | 0.24 (50) | 0 | 0 | 0.07 (15) |
| **Glioblastoma Multiforme** | 0 | 0.95 (304) | 0 | 0 | 0 | 0 | 0 | 0.05 (17) |
| **Head and Neck Squamous Cell Carcinoma** | 0.04 (25) | 0.04 (25) | 0 | 0.12 (81) | 0.03 (21) | 0 | 0.77 (512) | 0 |
| **Kidney Chromophobe** | 0.32 (30) | 0 | 0 | 0 | 0 | 0 | 0.68 (65) | 0 |
| **Kidney Renal Clear Cell Carcinoma** | 0.03 (27) | 0 (3) | 0 | 0 | 0 | 0 | 0.97 (929) | 0 |
| **Kidney Renal Papillary Cell Carcinoma** | 0.09 (26) | 0 | 0 | 0 | 0 | 0 | 0.91 (277) | 0 |
| **Liver Hepatocellular Carcinoma** | 0.1 (39) | 0 | 0 | 0 | 0 | 0 | 0.9 (357) | 0 |
| **Lung Adenocarcinoma** | 0 | 0.04 (23) | 0 | 0.17 (108) | 0 | 0 | 0.79 (503) | 0 |
| **Lung Squamous Cell Carcinoma** | 0 | 0.06 (29) | 0 | 0 | 0 | 0 | 0.94 (480) | 0 |
| **Diffuse Large B-cell Lymphoma** | 0.1 (5) | 0 | 0 | 0 | 0 | 0 | 0.9 (45) | 0 |
| **Mesothelioma** | 0 | 0 | 0 | 0 | 0 | 0 | 1 (82) | 0 |
| **Ovarian Serous Cystadenocarcinoma** | 0.04 (33) | 0.02 (14) | 0.93 (712) | 0 | 0 | 0 | 0 | 0.01 (9) |
| **Pancreatic Adenocarcinoma** | 0 | 0 | 0 | 0 | 0 | 0 | 1 (172) | 0 |
| **Pheochromocytoma and Paraganglioma** | 0 | 0 | 0 | 0 | 0 | 0 | 1 (174) | 0 |
| **Prostate Adenocarcinoma** | 0 | 0.02 (14) | 0 | 0.18 (108) | 0 | 0 | 0.8 (493) | 0 |
| **Rectum Adenocarcinoma** | 0.06 (18) | 0 | 0 | 0.14 (42) | 0 | 0 | 0.79 (231) | 0 |

| | | | | | | | | |
|---|---|---|---|---|---|---|---|---|
| **Sarcoma** | 0 | 0 | 0 | 0 | 0 | 0 | 0.89 (250) | 0.11 (30) |
| **Skin Cutaneous Melanoma** | 0 | 0.01 (1) | 0 | 0.12 (14) | 0 | 0 | 0.87 (102) | 0 |
| **Stomach Adenocarcinoma** | 0 | 0.04 (28) | 0.8 (573) | 0.15 (110) | 0.01 (5) | 0 | 0 | 0 |
| **Testicular Germ Cell Tumors** | 0 | 0 | 0 | 0 | 0 | 0 | 1 (133) | 0 |
| **Thymoma** | 0 | 0 | 0 | 0 | 0 | 0 | 1 (119) | 0 |
| **Thyroid Carcinoma** | 0 | 0.05 (28) | 0 | 0.11 (71) | 0.04 (23) | 0 | 0.8 (496) | 0 |
| **Uterine Carcinosarcoma** | 0 | 0 | 0 | 0 | 0 | 0 | 1 (57) | 0 |
| **Uterine Corpus Endometrial Carcinoma** | 0 | 0 | 0 | 0.12 (106) | 0 | 0 | 0.83 (723) | 0.04 (39) |
| **Uveal Melanoma** | 0 | 0 | 0 | 0 | 0.4 (51) | 0 | 0.6 (76) | 0 |

[1] Poore GD, Kopylova E, Zhu Q, Carpenter C, Fraraccio S, Wandro S, et al. Microbiome analyses of blood and tissues suggest cancer diagnostic approach. Nature. 2020;579:567-74.
